# On the possibility of hybrid chalcogenide perovskite photovoltaics

**DOI:** 10.1039/d6fd00025h

**Published:** 2026-07-14

**Authors:** Ruiqi Wu, J. J. Acton, Shirui Wang, Alex M. Ganose

**Affiliations:** a Department of Chemistry, Molecular Sciences Research Hub, White City Campus, Imperial College London Wood Lane London W12 0BZ UK a.ganose@imperial.ac.uk

## Abstract

Chalcogenide perovskites are an emerging class of photovoltaic absorbers offering stable, lead-free structures and promising optoelectronic properties. To date, the literature on chalcogenide perovskites has focused primarily on fully inorganic systems such as BaZrS_3_. This contrasts with the halide perovskites, for which hybrid organic–inorganic systems exhibit record performance. In this work, we assess the viability of hybrid chalcogenide perovskite absorbers using first-principles calculations. We screen a wide range of monovalent and divalent organic cations within the A-site to evaluate their electronic, optical, and thermodynamic properties. Our analysis reveals that the majority of candidates are structurally unstable; however, we identify the hydrazinium cation (N_2_H_6_^2+^) as a unique candidate that maintains a stable perovskite structure. Specifically, we identify N_2_H_6_ZrSe_3_ as the most promising candidate, exhibiting a quasi-direct band gap of 1.31 eV and a theoretical maximum efficiency of 24.5% for a 200-nm thin film. This study represents the first comprehensive computational report on hybrid chalcogenide perovskites, opening new avenues for the development of Earth-abundant photovoltaic materials.

## Abstract 2

First-principles screening of hybrid chalcogenide perovskites identifies hydrazinium-containing materials as stable solar absorbers. The optimal candidate, HZZrSe_3_, exhibits a 1.3 eV band gap and a theoretical maximum efficiency of 24.5%.

## Graphical abstract

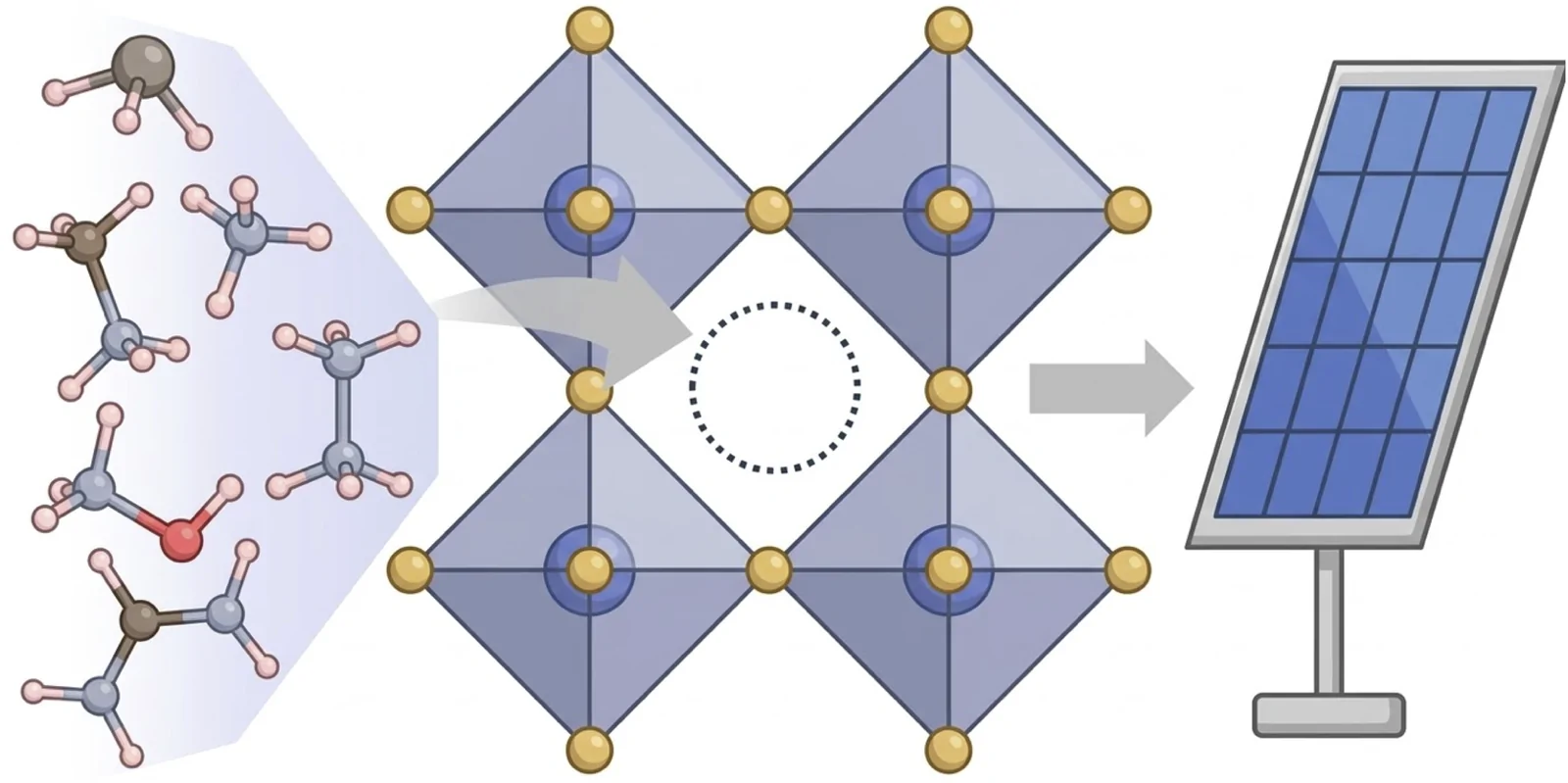

## Introduction

As solar energy deployment accelerates, the development of highly efficient, non-toxic, and Earth-abundant photovoltaic materials is paramount.^1^ While silicon dominates the current market, alternative thin-film technologies are often constrained by a reliance on toxic (*e.g.*, Cd, Pb) or scarce (*e.g.*, Te, In) elements, hindering their scalability.^2,3^ Over the last decade, perovskites have gained significant attention as a promising solution for next-generation photovoltaics. Their unique chemical and physical properties stem from the ABX_3_ crystal structure, characterised by a three-dimensional network of corner-sharing BX_6_ octahedra with an A-site cation occupying the cuboctahedral cavity.

The most widely researched perovskite photovoltaic devices are lead halide hybrid organic–inorganic perovskites, which comprise a monovalent organic A-site cation, a divalent Pb B-site cation, and a halide X-site anion.^4^ They are generally facile to synthesise using low-cost, solution-processable methods and are chemically tuneable, enabling high photovoltaic performance.^5,6^ To date, single-junction perovskite cells have reached record efficiencies of 26.7%.^7,8^ Methylammonium lead iodide (CH_3_NH_3_PbI_3_, or MAPbI_3_) is the most extensively studied perovskite absorber due to its exceptional optoelectronic properties. It displays a direct band gap of approximately 1.55 eV, low exciton binding energies, high defect tolerance, and remarkable charge carrier diffusion lengths.^9–12^ However, while hybrid lead halide perovskites continue to break efficiency records, their poor environmental stability and reliance on toxic lead have prevented wide-scale commercialisation.^13^

To address these challenges, chalcogenide materials, comprising group 16 anions (S^2−^, Se^2−^), have been integrated into the ABX_3_ perovskite architecture. This approach aims to combine the stability provided by strongly covalent metal–chalcogenide bonds with the favourable electronic dimensionality of the perovskite lattice.^14^ BaZrS_3_ is the most extensively studied chalcogenide perovskite. It crystallises in the orthorhombic GdFeO_3_-type (*Pnma*) structure and exhibits exceptional thermodynamic and environmental stability.^15^ The corner-sharing ZrS_6_ octahedra provide large band dispersion, leading to low effective masses and high charge carrier mobilities. Furthermore, BaZrS_3_ displays a high absorption coefficient (>10^5^ cm^−1^) near the absorption onset and a direct band gap of ≈1.78 eV.^15^ It is lead-free and several synthesis routes exist to form polycrystalline thin films.^16,17^ Crucially, chalcogenides such as BaZrS_3_ exhibit superior stability in the presence of moisture and elevated temperatures compared to their halide counterparts. Additionally, they benefit from reduced toxicity, a primary advantage over lead-halide- and cadmium-telluride-based photovoltaics.

To date, all experimental reports have focused on fully inorganic compounds. However, analogous to the halides, substituting the inorganic A-site with an organic molecule offers a route to tune optoelectronic properties, increase structural degrees of freedom, and potentially reduce synthesis costs. Currently, reports on hybrid chalcogenide perovskites remain scarce. Sun *et al.*^18^ theoretically investigated the substitution of the inorganic A-site with the organic hydrazinium ion (N_2_H_6_^2+^) to form [N_2_H_6_]ZrS_3_, predicting a direct band gap of 1.68 eV and a theoretical efficiency of 29% using the Heyd–Scuseria–Ernzerhof (HSE06) hybrid functional. *Ab initio* molecular dynamics at 423 K further suggested that the structure is dynamically stable.

In this work, we systematically assess the viability of hybrid organic–inorganic chalcogenide perovskites for photovoltaic applications using first-principles calculations. We screen a wide range of charge-balanced organic cations to identify those that yield geometrically stable perovskite structures. The identified candidates are relaxed to their ground state and their optoelectronic properties evaluated using hybrid density functional theory. We identify the HZ(Zr,Hf)(S,Se)_3_ family (where HZ = hydrazinium) as a unique candidate class, exhibiting band gaps within the ideal range for solar absorption. Subsequently, we assess the thermodynamic and dynamical stability of the most promising candidates using convex hull analysis and molecular dynamics. Finally, band alignment calculations are performed to identify suitable contact materials for device integration.

## Computational methodology

All calculations were performed using the Kohn–Sham DFT framework within the Vienna *Ab initio* Simulation Package (VASP), employing the projector augmented wave method.^19,20^ Structural optimisations utilised the generalised gradient approximation (GGA) of Perdew, Burke, and Ernzerhof,^21^ specifically the revised version adapted for solids (PBEsol).^22^ Atoms were relaxed until the Hellmann–Feynman forces were below 0.01 eV Å^−1^ and the total energy was converged to 1 × 10^−5^ eV. The plane-wave energy cut-off and *k*-point mesh were converged to tolerances of 5 meV per atom and 1 meV per atom, respectively, yielding a converged cut-off of 500 eV with the converged *k*-point meshes provided in SI Table S5. During geometry optimisations, the cut-off was increased to 650 eV to account for Pulay stress errors.^23^ The HSE06 functional was used to calculate the band structures, density of states (DOS) and optical absorption, which were plotted using the sumo package.^24,25^ Use of a hybrid functional is essential to correct for band-gap underestimation seen in semi-local GGA functionals, as previously demonstrated across many emerging photovoltaics.^26,27^ To further justify our choice of exchange correlation functionals, we benchmark their performance on the geometric and optoelectronic properties of BaZrS_3_ and BaHfS_3_ in Section S1 of the SI.

Structural stability and phase formation in perovskites are described by the Goldschmidt tolerance factor and octahedral factor, which quantify structural distortion and octahedral stability, respectively. The Goldschmidt tolerance factor (*t*)^28^ is defined as1
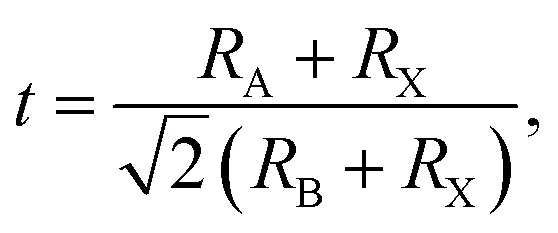
where *R*_A_, *R*_B_ and *R*_X_ are the ionic radii for the respective sites. A tolerance factor of 1 indicates preference for a cubic structure, while 0.8 < *t* < 1 is often considered a stable range for perovskites. The octahedral factor (*µ*) is a measure of whether the B-site cation is large enough to prevent the octahedron from collapsing and is satisfied when 0.4 ≤ *µ* ≤ 0.8. It is defined as2
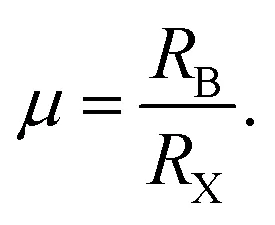
We derived the effective organic A-site cation radii, *r*_A_, following the approach of Kieslich *et al.*^29^ Assuming unrestricted rotation about the centre of mass, cations are treated as rigid spheres with

3 *r*_A_ = *r*_mass_ + *r*_ion_,

where *r*_mass_ represents the distance from the centre of mass to the most distant non-hydrogen atom and *r*_ion_ denotes the ionic radius of that specific atom.

To generate hybrid organic–inorganic chalcogenide perovskites, molecular substitution was implemented using the pymatgen^30^ package. Organic cations with oxidation states of +1 or +2 were used to replace the inorganic A-site atom, ensuring charge neutrality. Thermodynamic stability was evaluated using the doped^31^ software package, employing the convex hull formalism to assess stability relative to competing phases. Competing phases were identified by querying the Materials Project^32^ within the host chemical space and selecting phases that lie on the convex hull. For each target compound and its competing phases, the ground-state energies were calculated using a converged *k*-mesh and the PBEsol functional. The thermodynamic stability was determined by computing the energy above the hull of the target phase with respect to the competing phases, where an energy above the hull of 0 eV indicates stability, and a positive value implies a driving force to decompose into lower-energy phases.

*Ab initio* molecular dynamics (AIMD) simulations were performed on 2 × 4 × 4 supercells of the orthorhombic structure to assess the stability at finite temperature. Molecular dynamics simulations were performed in the isothermal–isobaric (NPT) ensemble using a Langevin thermostat with a Parrinello–Rahman barostat at 300 K, employing a timestep of 0.5 fs and a total simulation time of 10 ps. A single *k*-point at Γ was used for all AIMD simulations. To further reduce computational resources during AIMD simulations, we used on-the-fly machine-learning force fields (MLFF) employing Gaussian approximation potentials as implemented in VASP.^33,34^ The potentials were configured following the VASP 6.5.0 default settings. To identify the ground-state cation orientation, we performed an annealing strategy composed of an initial temperature ramp to 450 K, followed by an equilibration run at 450 K for 10 ps, and subsequent annealing to 10 K. All three stages were performed for 10 ps each, with a 0.5 fs timestep. The final structure at the end of the annealing run was relaxed using the trained on-the-fly forcefield, symmetrised using spglib,^35^ and relaxed again using PBEsol to obtain the final ground-state structure. Tilting-mode analysis was performed from molecular dynamics trajectories using the PDynA package.^36^

The high-frequency dielectric response (*ε*_∞_) was calculated using HSE06 through the frequency-dependent microscopic polarisability matrix as implemented in VASP.^37^ The low-frequency ionic dielectric response (*ε*_ionic_), which accounts for the response of phonons under an applied electric field, was calculated using density functional perturbation theory with the PBEsol functional. The static dielectric constant is given by *ε*_0_ = *ε*_∞_ + *ε*_ionic_. The conductivity effective mass, taking into account a Fermi–Dirac-weighted average of all bands near the band edge, was obtained using the AMSET package.^38^

To evaluate the upper radiative limit of thickness-dependent energy conversion, the Spectroscopic Limited Maximum Efficiency (SLME) detailed balance metric was employed, based on the optical properties calculated using HSE06.^39^ Band alignment calculations employed a slab structure generated using the surfaxe package^40^ with slab and vacuum thicknesses of 30 Å. The electron affinity and ionisation potentials were determined based on alignment between the core and vacuum levels of the slab, and core and valence band maximum of the bulk. All band alignment calculations were performed using the HSE06 functional.

## Results and discussion

### Materials screening strategy

To identify viable hybrid chalcogenide perovskites, we employed a hierarchical screening workflow, progressively filtering candidates based on chemical constraints, structural integrity, and thermodynamic stability. *Ab initio* molecular dynamics were employed to assess dynamical stability and capture cation-driven octahedral tilting. For stable compounds, we evaluate their optoelectronic properties relevant to photovoltaic operation, including band gaps, carrier effective masses, strength of optical absorption, and predicted maximum theoretical photovoltaic performance. Finally, band alignment analysis is performed to inform the selection of suitable contact layer materials for electron and hole extraction. The overall process is summarised in Fig. 1a.

**Fig. 1:**
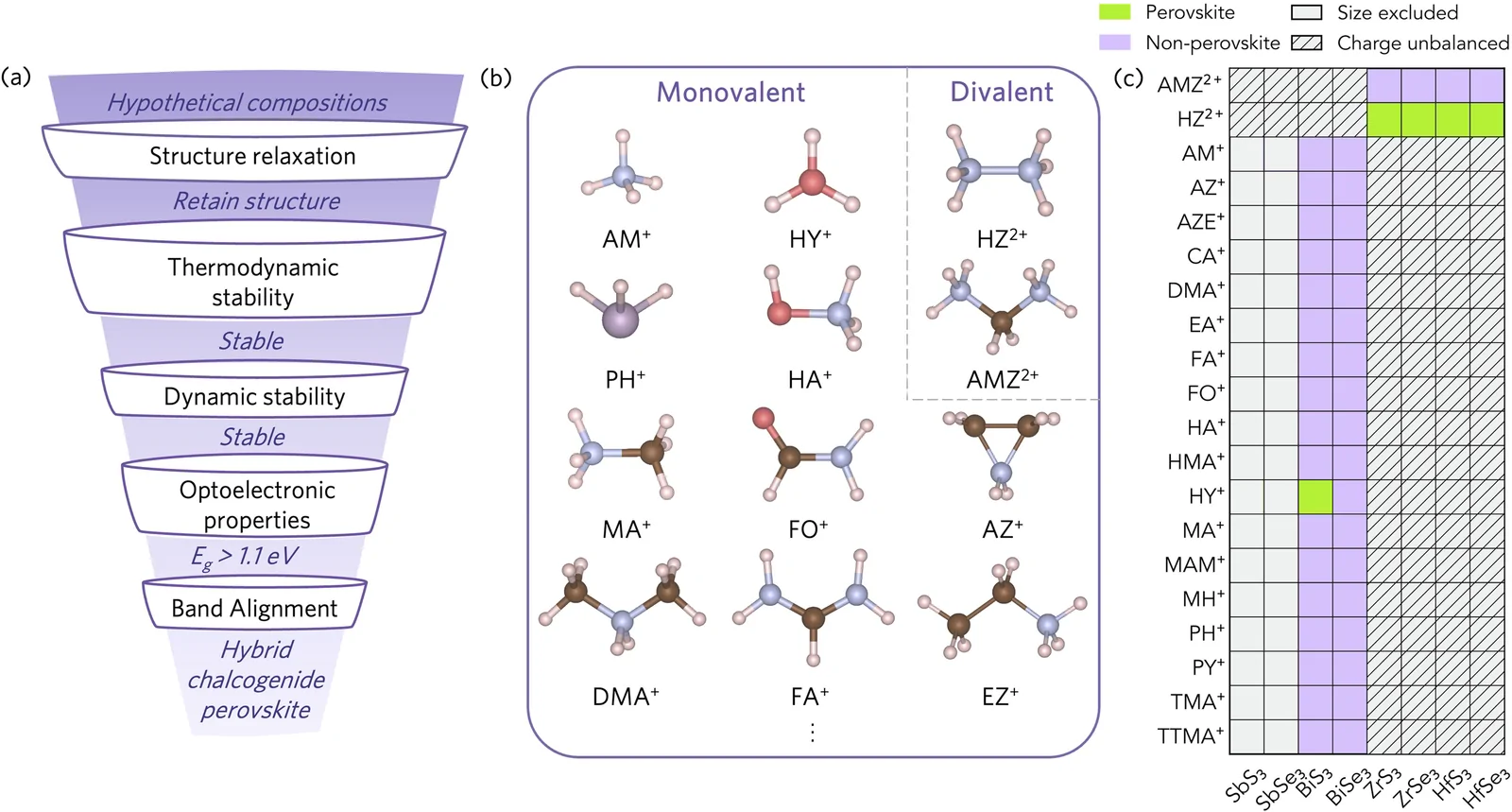
Overview of the material screening procedure used to identify the hybrid chalcogenide candidates from the 50 initial compounds. (a) Detailed screening workflow based on stability and optoelectronic properties. (b) The monovalent and divalent organic molecules studied in this work. (c) Structural stability after geometry optimisation using the PBEsol functional, where green and purple blocks refer to the perovskite and non-perovskite structures, respectively.

Our chemical search space was defined by the ABX_3_ perovskite stoichiometry. For the B-site, we identified two cation groups: pentavalent pnictogens (Sb, Bi) and tetravalent transition metals (Zr, Hf). These elements were prioritised due to their relatively high Earth abundance, reduced toxicity compared to Pb or Cd, and their use in emerging chalcogenide absorbers such as Sb_2_Se_3_ and BaZrS_3_.^41^ The choice of B-site dictates the charge requirements for the organic A-site to maintain neutrality. The group 15 series (Sb, Bi) requires a monovalent (A^+^) cation, while the group 4 series (Zr, Hf) requires a divalent (A^2+^) cation.

To begin, a stable ground-state bulk structure is required as a reference framework into which the A-site cation can be substituted. Given that perfect cubic structures (*e.g.*, SrTiO_3_) are rare in later-group chalcogenide perovskites, we used the orthorhombic BaZrS_3_ ground-state phase (*Pnma*) as a reference structure.^14^ The initial structure was obtained from the Materials Project database.^32^ Candidate organic cations were identified by querying the PubChem database^42^ for molecules satisfying the charge neutrality requirements imposed by the B-site monovalent cations for Sb/Bi and divalent cations for Zr/Hf. We applied a molar mass cut-off of 75 g mol^−1^ as a proxy for ionic size to maximise the probability of fitting within the steric constraints of the chalcogenide cage. The full set of organic cations considered is provided in Table 1.

**Table 1:** Organic cations used as A-site molecules in the ABX_3_ perovskite structure, including the molar mass (*M*) in g mol^−1^. The cation radius was calculated according to eqn (3)

Cation	Formula	Label	Molar mass (g mol^−1^)	*R*_A_ (pm)
Ammonium	[NH_4_]^+^	AM^+^	18.04	146
Hydronium	[H_3_O]^+^	HY^+^	19.02	220
Methaniminium	[CH_2_NH_2_]^+^	MAM^+^	30.05	216
Methylammonium	[CH_3_NH_3_]^+^	MA^+^	32.06	217
Hydrazinium	[NH_3_NH_2_]^+^	HZ^+^	33.05	220
Hydroxylammonium	[NH_3_OH]^+^	HA^+^	34.04	216
Phosphonium	[PH_4_]^+^	PH^+^	35.00	212
Aziridinium	[NH_2_(CH_2_)_2_]^+^	AZ^+^	44.08	238
Formamidinium	[CH(NH_2_)_2_]^+^	FA^+^	45.08	253
Ethylammonium	[CH_3_CH_2_NH_3_]^+^	EA^+^	45.10	274
Formamide	[NH_3_COH]^+^	FO^+^	46.06	258
Dimethylazanium	[NH_2_(CH_3_)_2_]^+^	DMA^+^	46.09	272
Methylhydrazinium	[CH_3_NH_2_NH_3_]^+^	MH^+^	47.08	275
Azetidinium	[NH_2_(CH_2_)_3_]^+^	AZE^+^	58.10	250
Hydroxy(methylidene)azanium	[CH_2_NHOH]^+^	HMA^+^	58.06	275
Cyclopropylammonium	[NH_3_CH(CH_2_)_2_]^+^	CA^+^	58.10	293
Trimethylazanium	[NH(CH_3_)_3_]^+^	TMA^+^	60.12	294
Pyrrolidinium	[NH_2_(CH_2_)_4_]^+^	PY^+^	72.13	278
Tetramethylazanium	[N(CH_3_)_4_]^+^	TTMA^+^	74.14	292
Hydrazinium	[NH_3_NH_3_]^2+^	HZ^2+^	34.06	217
Azaniumylmethylazanium	[CH_2_(NH_3_)_2_]^2+^	AMZ^2+^	48.11	267

Sourcing suitable divalent organic cations presented a significant chemical challenge, since they are often intrinsically unstable due to electron deficiencies and high ionisation energies. Consequently, only two realistic divalent candidates were identified: hydrazinium (NH_3_–NH_3_)^2+^ and azaniumylmethylazanium (NH_3_–CH_2_–NH_3_)^2+^. Due to this scarcity, monovalent cations dominate our list as they are far more abundant (see Table 1). In total, we identified 84 unique hypothetical compositions for evaluation. As illustrated in the matrix in Fig. 1c, the combination of these species defines the screening phase space. The hatched grey blocks correspond to charge-imbalanced compositions. The remaining grey blocks represent size-excluded compositions, which we define as compounds with an octahedral factor less than 0.34. This resulted in all of the Sb compounds being screened out, with a final set of 46 compositions selected for further study. The full list of tolerance and octahedral factors for all 84 compounds is provided in Table S4 of the SI.

### Structural stability

The structural viability of the 46 hypothetical compounds was assessed *via* geometry optimisation using the PBEsol functional. A candidate was considered structurally stable only if it retained the corner-sharing BX_6_ octahedral network characteristic of the perovskite phase upon relaxation. As shown by the purple blocks in Fig. 1c, the majority of compounds do not produce a stable perovskite structure, instead forming non-perovskite phases due to bond breaking resulting from large lattice distortions.

In the monovalent A^+^Bi^5+^X_3_ series, structural instability was pervasive. As previously discussed, only Bi was considered as the B-site cation due to its larger ionic radius (0.76 Å) compared to Sb (0.60 Å) in the 5^+^ charge state, increasing the octahedral factor closer to 0.40.^43^ Among the monovalent A-site cations, only hydronium (HY^+^) retained the corner-sharing perovskite framework. This cation has a small effective radius (*R*_A_ = 140 pm) and low molecular weight, which favours accommodation within the A-site cavity, consistent with results reported by Park *et al.*^44^ In contrast, structural stability declines sharply for effective radii exceeding 220 pm, with no other monovalent cations remaining stable in the perovskite structure.

In the divalent A^2+^B^4+^X_3_ series (B = Zr, Hf), we observed a strict size cut-off. The azaniumylmethylazanium compounds (AMZ^2+^), containing a methylene bridge (–CH_2_–) between the amine groups, decomposed into low-energy non-perovskite structures. The only survivor was the hydrazinium cation (N_2_H_6_^2+^, HZ), which lacks the methylene spacer and possesses a smaller effective radius of 217 pm. This can be seen in Table 2. The HZ cation successfully retained the orthorhombic perovskite structure for both Zr- and Hf-based compounds.

**Table 2:** Effective organic cation radius (*R*_A_) and ionic radii (*R*_B_, *R*_X_) used to calculate the tolerance factor (*t*) and octahedral factor (*µ*), as well as the average octahedral tilting angle for the chalcogenide perovskites

Compound	*R*_A_ (pm)	*R*_B_ (pm)	*R*_X_ (pm)	*t*	*µ*	Tilt angle (°)
HYBiS_3_	140	76	184	0.90	0.41	3.08
HZZrS_3_	217	72	184	1.11	0.39	9.72
HZHfS_3_	217	71	184	1.11	0.39	9.11
HZZrSe_3_	217	72	198	1.09	0.36	11.86
HZHfSe_3_	217	71	198	1.09	0.36	11.30

All surviving perovskite structures exhibited Goldschmidt tolerance factors in the range 0.90 < *t* < 1.11, consistent with a distorted pseudocubic or orthorhombic coordination,^28^ and an octahedral factor *µ* > 0.36. Although an octahedral factor of >0.40 is desirable, compounds can remain stable with values slightly below this threshold, as highlighted by BaZrS_3_ (*µ* = 0.39).

### Thermodynamic stability

Structural preservation after relaxation is only a weak proxy for stability. The material must also be thermodynamically stable against decomposition into competing phases. We evaluated the thermodynamic stability of the structurally surviving candidates by calculating their energy above the convex hull (*E*_hull_) using PBEsol. Thermodynamic properties were analysed based on formation energies and stability against competing phases, where negative formation energies indicate stability with respect to elemental ground states.^45^

The monovalent HYSbS_3_ exhibited a large *E*_hull_ value of over 400 meV per atom, indicating thermodynamic instability. Despite retaining the perovskite geometry, this material is expected to decompose into lower-energy binary chalcogenides and volatiles under any realistic synthesis conditions. Consequently, none of the monovalent candidates pass both the thermodynamic and structural stability checks, and the entire monovalent series was excluded from further consideration.

In contrast, the divalent HZ-based family (HZZrS_3_, HZZrSe_3_, HZHfS_3_, HZHfSe_3_) was found to lie on the convex hull (*E*_hull_ = 0 eV per atom). This confirms that these hybrid hydrazinium chalcogenides represent the thermodynamic ground states for their respective compositions, suggesting they could be synthetically accessible. Based on this screening process, only the HZ family was selected for detailed optoelectronic characterisation.

### Dynamic stability

To assess the robustness of the perovskite framework at finite temperatures, we performed MLFF-accelerated molecular dynamics simulations. Fig. 2a presents the evolution of the cell volume for the HZZrS_3_, HZZrSe_3_, HZHfS_3_, and HZHfSe_3_ compounds over a 10 ps trajectory at 300 K. In all cases, the volume fluctuates around a constant equilibrium value with no drift. This indicates the absence of phase transitions or decomposition pathways, further suggesting that the hybrid hydrazinium chalcogenides are dynamically stable at room temperature.

**Fig. 2:**
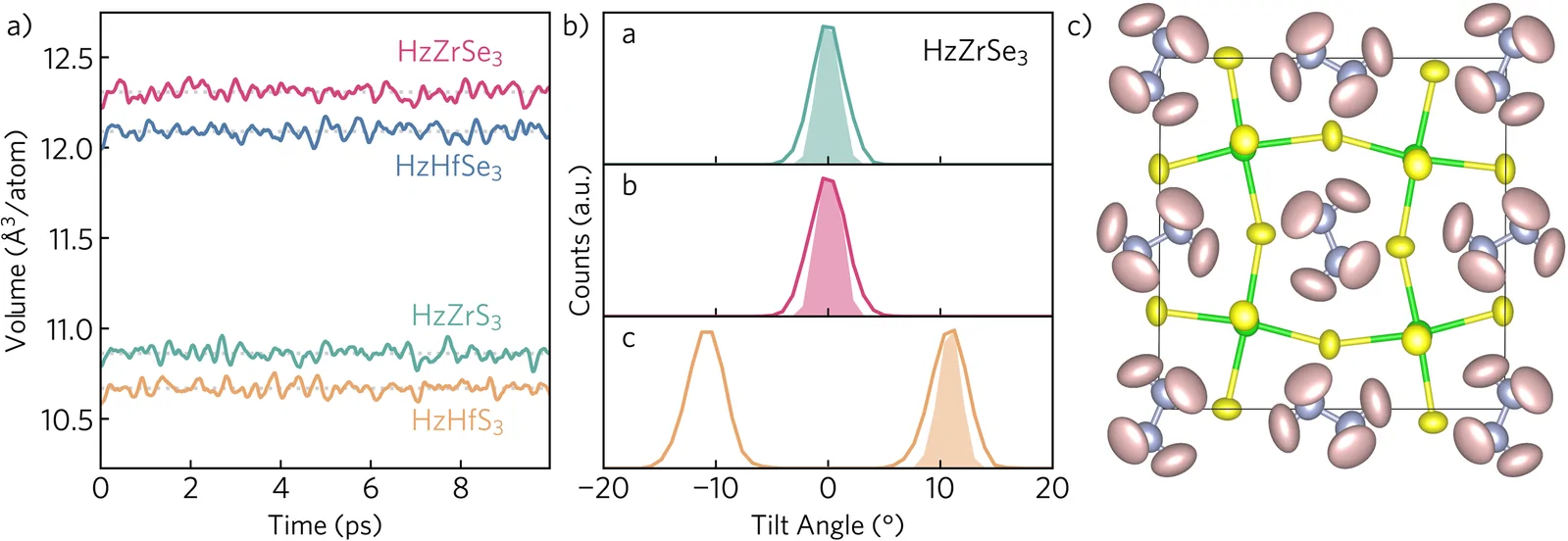
Dynamical stability and structural analysis of hydrazinium chalcogenide perovskites at 300 K. (a) Time evolution of the volume per atom for HZZrS_3_, HZZrSe_3_, HZHfS_3_, and HZHfSe_3_ over a 10 ps MLFF-MD trajectory, indicating structural stability. (b) Dynamic distribution of octahedral tilt angles for HZZrSe_3_ decomposed into the principal lattice vectors. Solid lines show the distribution of tilting, while the shaded region represents the corresponding nearest-neighbour tilts along the same direction. The shaded area in the positive angle range reveals an in-phase correlation along the *c*-axis (*a*^0^*b*^0^*c*^+^ in Glazer notation). (c) Thermal ellipsoid plot of HZZrSe_3_ derived from the MD trajectory (80% probability), shown in the *a*–*b* plane.

To further understand the dynamics of the octahedral framework and organic cations, and gain insight into the local symmetry breaking, we extracted the instantaneous octahedral tilting angles (*θ*) and their spatial correlations from the MD trajectories using the PDynA package.^36^ As shown in the histogram for HZZrSe_3_ (Fig. 2b), the tilting behaviour is highly anisotropic. Distributions for the *a* and *b* axes are centred at zero, indicating no permanent static tilt along these directions. In contrast, the *c*-axis exhibits a distribution with two peaks with a mean magnitude of 11.38° (averaged values for all compounds are detailed in Table 2). Crucially, the spatial correlation function along the *c*-axis is positive, indicating an in-phase rotation of adjacent octahedra. This tilting pattern corresponds to the *a*^0^*b*^0^*c*^+^ Glazer system,^46^ which lowers the symmetry of the aristotype structure to the orthorhombic *Pbam* space group. In the fully inorganic analogue, the same *a*^0^*b*^0^*c*^+^ tilt system is typically associated with the tetragonal *P*4/*mbm* space group, while A-site molecular ordering and hydrogen bonding in the hybrid chalcogenide introduce additional symmetry breaking, lowering the overall symmetry to *Pbam*. This is in contrast to BaZrS_3_, which crystallises in the *Pnma* structure characterised by a complex three-axis tilting system (*a*^−^*b*^+^*a*^−^).^15^ The reduction in tilting dimensionality in the hybrid systems suggests that the anisotropic shape and steric bulk of the hydrazinium cation effectively suppress rotations along the in-plane axes. The full set of lattice parameters for the relaxed stable chalcogenide perovskites are provided in SI Table S6.

The limited rotation of the hydrazinium cation is further elucidated by the thermal displacement analysis presented in Fig. 2c for HZZrSe_3_. The thermal ellipsoids for the Zr and Se sites are relatively small, consistent with a stiff inorganic framework with limited degrees of rotation. For the organic A-site, the nitrogen atoms of the hydrazinium molecule also exhibit small thermal ellipsoids. While the hydrogen atoms display larger displacement parameters, the anchoring of the N–N axis indicates that the molecule is fixed by a hydrogen-bonding network with the chalcogen anions, as seen at low temperatures in other hybrid perovskite compounds. This further highlights that the N_2_H_6_^2+^ molecule is not rotationally disordered but is instead locked into a specific orientation within the cuboctahedral cavity, in contrast to the methylammonium cation in halide perovskites (MAPbI_3_), which display considerably more rotational degrees of freedom at room temperature.^47^

### Electronic properties

Having established the thermodynamic and dynamic stability of the hydrazinium-based perovskites, we now turn to their electronic structure to assess their viability as photovoltaic absorbers. Fig. 3 presents the crystal structure and electronic band structure of HZZrS_3_ calculated using the HSE06 hybrid functional. The corresponding data for the isoelectronic and isostructural analogues (HZZrSe_3_, HZHfS_3_, and HZHfSe_3_) are provided in SI Fig. S5 and display similar features.

**Fig. 3:**
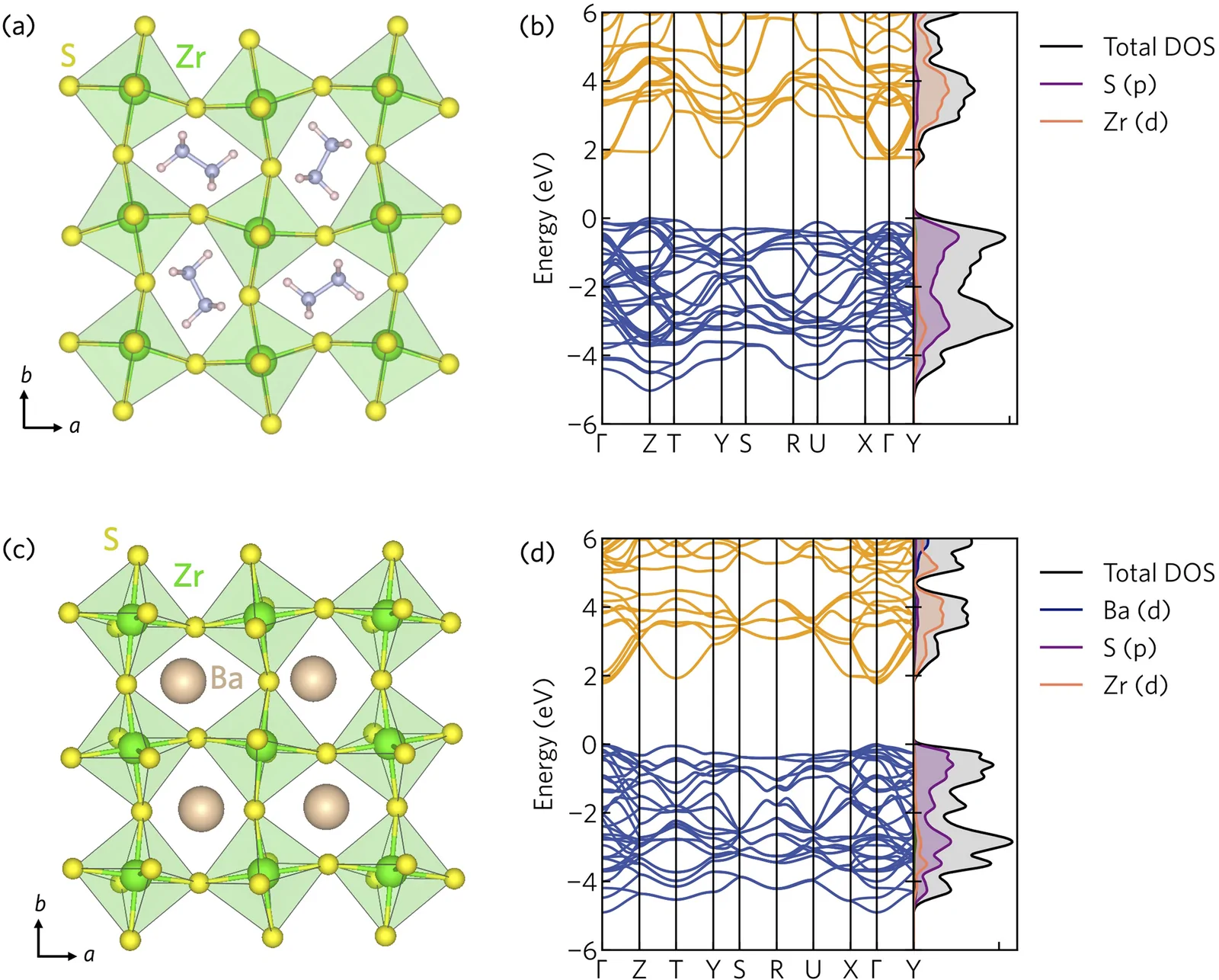
Comparison of crystal structure and electronic properties between hybrid and inorganic chalcogenide perovskites. (a) Crystal structure and (b) band structure of HZZrS_3_. (c) Crystal structure and (d) band structure of BaZrS_3_.

The calculated density of states (DOS) indicates that the optoelectronic behaviour of the HZBX_3_ series is governed almost exclusively by the inorganic framework. The valence band (VB) edge is dominated by chalcogen p states, while the conduction band (CB) is composed primarily of B-site transition metal d orbitals. Crucially, the N s and p orbitals of the hydrazinium cation make negligible contributions to the frontier states. This confirms that the organic moiety does not participate directly in charge transport. Instead, its role is structural, controlling the degree of lattice distortion and ensuring charge neutrality, with indirect impacts on electronic properties. This behaviour is consistent across the series, although some Hf p contributions are observed deep in the valence band (−4 eV) for the Hf analogues, reflecting their semi-core nature.

A key consequence of the hybrid A-site substitution is a modification of the band-edge momentum. As illustrated in Fig. 3, while the inorganic BaZrS_3_ is a direct-gap semiconductor, the hybrid sulphide compounds (HZZrS_3_ and HZHfS_3_) exhibit an indirect fundamental gap, with the valence band maximum (VBM) located at the Z-point and the conduction band minimum (CBM) at the Γ-point. We attribute this transition to the specific symmetry-breaking distortions induced by the anisotropic HZ^2+^ cation. In the inorganic BaZrS_3_ structure (*Pnma*), the octahedral tilting pattern preserves the VBM at Γ. However, the hybrid HZZrS_3_ structure adopts a distinct tilting mode driven by the rigid, hydrogen-bonded hydrazinium network. This alters the orbital overlap in the (001) plane, enhancing the antibonding S 3p–S 3p interactions at the Z-point relative to the inorganic phase. This interaction pushes the valence band energy at the Z-point above that at Γ, resulting in an indirect transition. Crucially, the magnitude of this indirectness (Δ*E*_Γ–Z_) is small and composition-dependent (Fig. 4a). For HZZrS_3_, the direct transition at Γ is only 0.12 eV higher than the fundamental indirect gap. Substituting S with Se significantly reduces this difference to just 0.05 eV in HZZrSe_3_, rendering the band gap effectively direct at room temperature due to thermal broadening. Conversely, the Hf analogues exhibit slightly larger differences, with HZHfS_3_ and HZHfSe_3_ showing Δ*E*_Γ–Z_ values of 0.15 eV and 0.11 eV, respectively.

**Fig. 4:**
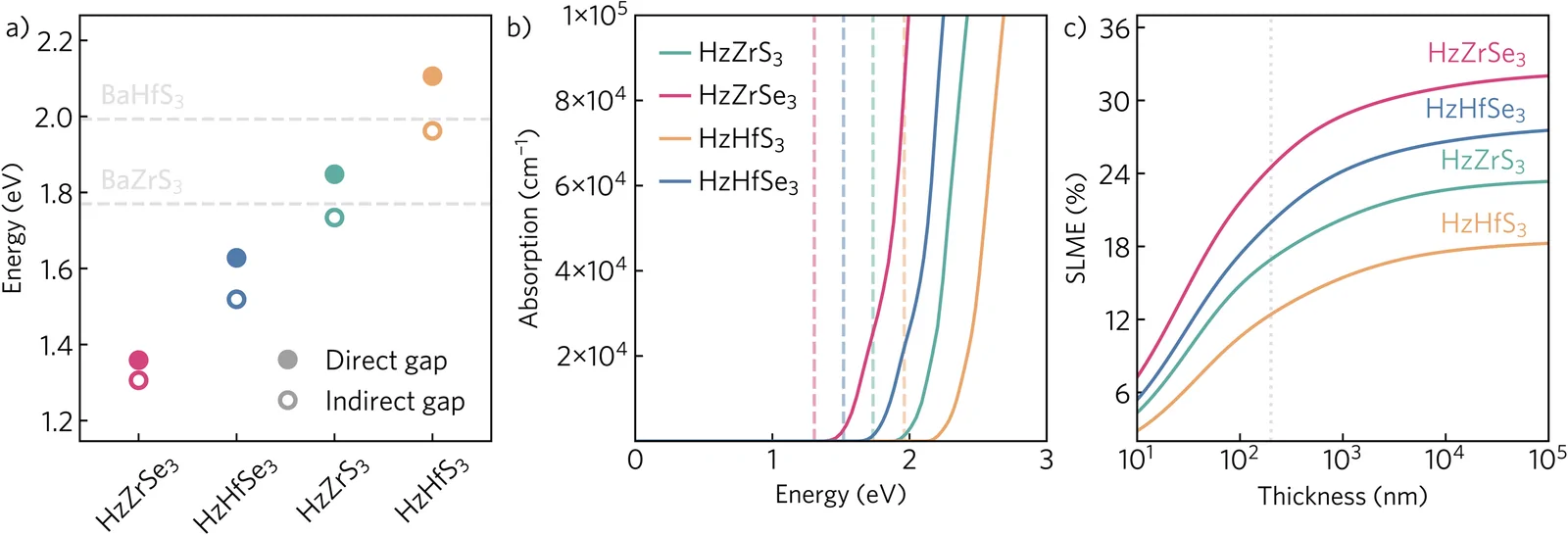
Optoelectronic properties of hybrid chalcogenide perovskites calculated using HSE06. (a) Direct (filled circles) and indirect (open circles) band gaps of the series. (b) Optical absorption of the compounds as a function of energy. The dashed coloured lines represent the indirect-band-gap energy. (c) Spectroscopic Limited Maximum Efficiency (SLME) metric for the compounds as a function of film thickness. The dashed grey line represents a thickness of 200 nm as a guide to the eye.

Despite their indirect nature, the magnitude of the band gaps remains favourable for single-junction photovoltaics (Table 3). The band gaps follow a clear chemical trend: HZHfS_3_ (2.11 eV) > HZZrS_3_ (1.85 eV) > HZHfSe_3_ (1.63 eV) > HZZrSe_3_ (1.31 eV). The substitution of S for Se systematically narrows the band gap, driven by the higher energy (lower ionisation potential) of the more diffuse Se 4p orbitals which raises the VBM. Conversely, substituting Zr (4d) with Hf (5d) widens the gap by approximately 0.2 eV, consistent with the higher orbital energy of the Hf 5d orbitals which raises the CBM.

**Table 3:** Optoelectronic properties of hydrazinium-based compounds. The direct (*E*^d^_g_) and indirect (*E*^i^_g_) band gaps are given in eV, the effective masses (*m*_e_ and *m*_h_) are given in units of the electron rest mass. We include the high-frequency (*ε*_∞_) and static dielectric constants (*ε*_0_) along the *a*, *b*, and *c* lattice vectors. The corresponding theoretical efficiency at 200 nm (*η*_200_) is also provided. The ionisation potential (IP) and electron affinity (EA) are given in eV

Compound	*E* ^d^_g_ (eV)	*E* ^i^_g_ (eV)	*m*_e_ (*m*_0_)	*m*_h_ (*m*_0_)	*ε*_∞_			*ε*_0_			*η*_200_ (%)	EA (eV)	IP (eV)
					*a*	*b*	*c*	*a*	*b*	*c*			
HZZrS_3_	1.85	1.73	0.58	1.59	6.6	6.8	6.9	80.8	71.3	88.2	16.9	4.34	6.07
HZZrSe_3_	1.36	1.31	0.56	1.17	8.2	8.6	7.8	82.8	110.8	108.7	24.5	4.53	5.84
HZHfS_3_	2.11	1.96	0.55	1.52	6.2	6.4	6.4	50.2	51.5	52.3	12.4	4.14	6.11
HZHfSe_3_	1.77	1.77	0.33	1.31	7.5	7.8	7.3	64.4	69.9	73.4	20.0	4.37	5.89

To provide an indication of carrier transport under realistic operating conditions, we calculated conductivity effective masses using the AMSET package.^38^ Unlike simple parabolic band fitting, this approach accounts for band non-parabolicity and multi-valley effects by computing a weighted average of the group velocities at a carrier concentration of 10^18^ cm^−3^ and 300 K. This formalism is essential for the hybrid chalcogenides since the symmetry-breaking octahedral tilting introduces a flat band near the conduction band minimum, a new feature that is absent in the inorganic BaZrS_3_. The presence of this heavy band, lying nearly degenerate with the conduction band minimum, increases the electron effective masses of the hybrid series (0.51–0.58*m*_0_) relative to inorganic BaZrS_3_ (0.33*m*_0_). For holes, the effective masses range from 1.17–1.59*m*_0_ (Table 3). Notably, the selenides exhibit lighter holes than both their sulphide counterparts and the inorganic BaZrS_3_ reference (1.31*m*_0_), driven by the increased bandwidth afforded by the Se 4p orbitals. Overall, these results indicate that while the hybrid A-site induces charge localisation, the resulting transport properties remain sufficient for efficient carrier collection and reduced recombination losses in thin-film device architectures. In particular, HZZrSe_3_ stands out with an ideal quasi-direct band gap value of 1.31 eV and reasonable effective mass for both electron and holes.

### Optical properties and predicted photovoltaic performance

While favourable band gaps and carrier effective masses are a prerequisite for candidate absorbers, high photovoltaic performance ultimately depends on the material’s ability to absorb light and generate voltage. Fig. 4b presents the optical absorption spectra for the series calculated using the HSE06 hybrid functional. Despite the indirect nature of the fundamental band gap, the absorption onsets are strong, exceeding 10^5^ cm^−1^ within 0.5 eV of the indirect gap. This steep rise is a direct consequence of the small energy difference between the fundamental direct/indirect transitions and the presence of localised states at the valence band edge. While weak phonon-assisted absorption is not explicitly modelled in our simulations, this will be almost immediately overtaken by strong, dipole-allowed direct transitions. This behaviour contrasts favourably with silicon, where the large difference between indirect and direct gaps necessitates thick absorber layers to ensure sufficient absorption.

To quantify the theoretical photovoltaic performance limit, we calculated the Spectroscopic Limited Maximum Efficiency (SLME) as a function of film thickness (Fig. 4c).^39^ This metric accounts for the absorption of the material and the thermodynamic losses associated with an indirect band gap. We note that non-radiative recombination is not treated explicitly and therefore this metric represents a maximum theoretical performance in the absence of other efficiency loss mechanisms. Consistent with their band gaps, the selenide compounds exhibit higher potential efficiencies than the sulphides. HZZrSe_3_ yields the highest theoretical efficiency of 24.5% for a 200-nm film, followed by HZHfSe_3_ at 20.0%. We note that our calculated efficiency for HZZrS_3_ (16.9%) is lower than the 29% reported previously by Sun *et al.*^18^ This difference is attributed to the omission of the indirect band gap in their calculations, which leads to a higher overall performance prediction, along with reporting the maximum efficiency at very large thicknesses. Indeed, we calculate a maximum theoretical efficiency of 29.5% when only the direct band gap is considered. The predicted efficiency of HZZrSe_3_ significantly outperforms other emerging antimony chalcogenide absorbers such as Sb_2_Se_3_ (∼10%)^41^ at comparable thicknesses.

Finally, we consider the dielectric response, which plays a crucial role in charge carrier screening and defect tolerance. Table 3 summarises the high-frequency (*ε*_∞_) and static (*ε*_0_) dielectric constants. The introduction of the hydrazinium cation and the associated lattice distortion leads to significant anisotropy. The high-frequency dielectric constants range from 6.2 to 8.6, comparable to established thin-film absorbers like CdTe (*ε*_∞_ ≈ 7.1).^48^ The static dielectric constants are considerably larger, ranging from 50 to 110. These values are substantially higher than those of typical inorganic chalcogenides and suggest effective dielectric screening of charged defects and impurities.^49^ Such strong screening typically suppresses carrier capture rates and reduces the binding energy of excitons, facilitating efficient charge separation and transport even in the presence of defects.

### Band alignments

Finally, to inform the design of suitable device architectures, we calculated the band positions relative to the vacuum level. Fig. 5 summarises the ionisation potentials (IP) and electron affinities (EA) for the HZ series. We find that the band positions are relatively deep compared to established absorbers, a consequence of the high electronegativity of S and Se. The ionisation potentials range from 5.8 eV to 6.1 eV. We observe a clear chemical trend where the sulphide compounds exhibit deeper valence bands compared to their selenide counterparts. This ∼0.3 eV shift is driven by the lower binding energy of the Se 4p orbitals relative to the S 3p orbitals, which pushes up the valence band maximum. This trend is beneficial for device integration, as the shallower IP of the selenides facilitates hole extraction. Conversely, the conduction band positions are governed by the B-site cation. Hf-based compounds exhibit slightly shallower electron affinities (HZHfS_3_: 4.1 eV) than their Zr-based analogues (HZZrS_3_: 4.3 eV), consistent with the higher orbital energy of the Hf 5d orbitals. The values are broadly comparable to the inorganic chalcogenide perovskites, for example, BaZrS_3_ (IP = 6.3 eV; EA = 4.6 eV),^50^ and the mixed anion absorber BiSI (IP = 6.4 eV; EA = 4.9 eV).^51^

**Fig. 5:**
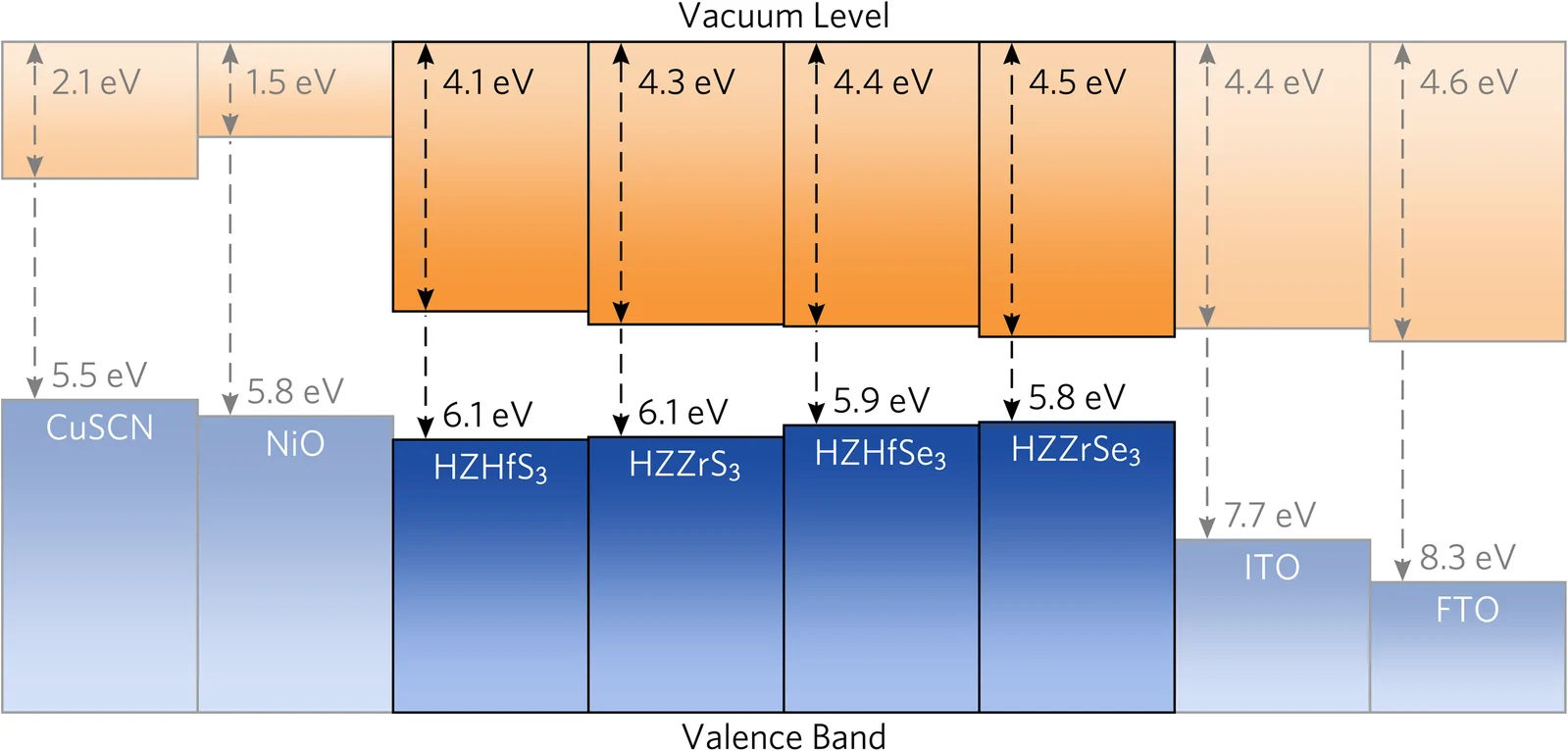
Comparison of absolute band alignments for hybrid chalcogenide perovskites against other well-known and emerging photovoltaic absorbers.

The electron affinities (4.1 eV to 4.5 eV) are well-matched to common n-type metal oxide transport layers. For instance, SnO_2_ (EA ≈ 4.5 eV)^52^ and indium tin oxide (EA ≈ 4.4 eV)^53,54^ are likely to form favourable ohmic contacts for electron extraction, particularly for the Zr-based compounds. However, hole extraction poses a more significant challenge. With ionisation potentials exceeding 6.0 eV, common hole-transport materials such as Spiro-OMeTAD or P3HT (IP ≈ 5.0 eV)^55,56^ would create large interfacial barriers, blocking hole transport and limiting the maximum open-circuit voltage. Instead, high-work-function inorganic hole transporters will be required. Likely candidates include NiO_*x*_ (IP ≈ 5.4 eV to 6.0 eV)^57^ or CuSCN (IP ≈ 5.5 eV),^58^ although interface engineering may still be needed to minimise voltage losses.

## Conclusions

In this work, we have employed first-principles calculations to systematically assess the viability of hybrid chalcogenide perovskites as stable, lead-free alternatives for thin-film photovoltaics. Through a hierarchical screening of 21 organic A-site cations, we identified the hydrazinium family, HZ(Zr,Hf)(S,Se)_3_, as the only candidates capable of supporting a stable perovskite framework. Thermodynamic analysis and molecular dynamics simulations suggest that these compounds are stable against decomposition and maintain dynamical stability at room temperature. Furthermore, we demonstrate that while these systems possess indirect fundamental band gaps, the degree of indirectness is small (0.05 eV to 0.15 eV), rendering these materials effectively quasi-direct absorbers at room temperature. This is reflected in their optical properties, which exhibit absorption coefficients exceeding 10^5^ cm^−1^ just above the band edge and yield theoretical efficiencies of up to 24.5% for 200-nm HZZrSe_3_ films. Furthermore, despite the presence of a flat band introduced by octahedral tilting, the carrier effective masses remain sufficiently low to support efficient charge transport.

The primary challenge for hybrid chalcogenide perovskites lies in their chemical constraints and device integration. The strict steric requirement for small A-site cations (*R*_A_ < 217 pm) limits the chemical space available for tuning, while the deep ionisation potentials (∼6 eV) necessitate the development of high-work-function hole-contact materials. Furthermore, since these are purely hypothetical compounds, a major challenge will be identifying feasible synthesis routes. We propose a solution-based route utilising commercially available hydrazinium sulphate (N_2_H_6_SO_4_) as a precursor:^59^

4 [N_2_H_5_][N_3_] + H_2_SO_4_ → N_2_H_6_SO_4_ + NH_3_

This salt can be used in a solid-state reaction with zirconium disulphide (ZrS_2_) and hydrogen sulphide (H_2_S) to synthesise HZZrS_3_, analogous to existing chalcogenide synthesis methods:^60–62^

5 N_2_H_6_SO_4_ + ZrS_2_ + H_2_S → N_2_H_6_ZrS_3_ + H_2_SO_4_

If these synthetic and interfacial challenges can be addressed, hybrid hydrazinium cations could offer a new direction for increasing the tunability of chalcogenide perovskite photovoltaics.

## Conflicts of interest

There are no conflicts to declare.

## Supplementary Material

FD-268-D6FD00025H-s001

## Data Availability

Raw calculation data for this article, including DFT input and output files, are available on NOMAD at https://doi.org/10.17172/nomad.rnsk-txjx. All code for generating the analysis presented in this work is made available on GitHub at https://github.com/virtualatoms/HybridChalcogenidePerovskites. Supplementary information (SI) is available. See DOI: https://doi.org/10.1039/d6fd00025h.
