# Supplementary material for: On the possibility of hybrid chalcogenide perovskite photovoltaics
Source: Faraday Discuss. 2026 Jul 14;268:77–94. doi: 10.1039/d6fd00025h (PMC13719144; doi:10.1039/d6fd00025h)
Supplement: Supplementary file 1 [file FD-268-D6FD00025H-s001.pdf]

Supplementary Information (SI) for Faraday Discussions.
This journal is © The Royal Society of Chemistry 2026

Supplementary Information: On the possibility of hybrid chalcogenide perovskite photovoltaics

S1 Inorganic Chalcogenide Perovskites

This section presents the results for two inorganic chalcogenide perovskites, $BaZrS_3$ and $BaHfS_3$, selected due to their prior experimental validation.[1–5] Comparing the computational results in this study with established experimental data serves to validate the accuracy of our calculation parameters. Once verified, these parameters can be applied to hybrid compounds to predict the properties of novel chalcogenide perovskites lacking experimental confirmation, ensuring the reliability and precision of the computational methods employed. Table S1 shows the converged properties for the inorganic chalcogenides mentioned ($BaZrS_3$ and $BaHfS_3$), adhering to the geometric and electronic parameters described in the main text.

Table S1: Calculation parameters for $BaZrS_3$ and $BaHfS_3$.

Compound	Structure	Space group	Cut-off (eV)	k-point mesh
$BaZrS_3$	Orthorhombic	*Pnma*	300	$3 \times 3 \times 4$
$BaHfS_3$	Orthorhombic	*Pnma*	300	$4 \times 3 \times 4$

The geometries of these structures were optimised using the PBEsol functional. The associated lattice parameters are listed in Table S2. In both compounds, the calculated lattice parameters are within 1% of experimental values, confirming the parameters are suitable for subsequent use on theoretical compounds. Figure S1 shows the optimised structures of

$BaZrS_3$ and $BaHfS_3$. Both chalcogenide perovskites exhibit $a^-b^+a^-$ octahedral tilting in the orthorhombic phase, reducing symmetry and altering bond angles and lengths.

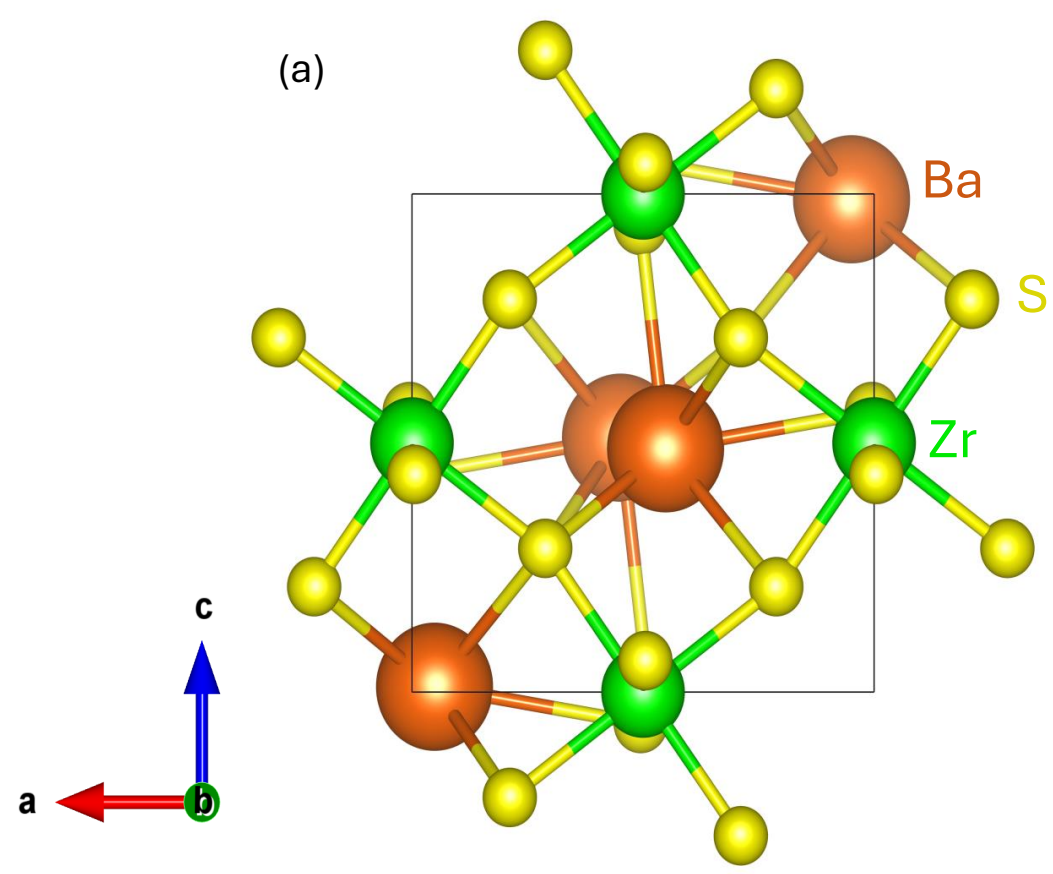

Figure S1: Optimised structure of (a) $BaZrS_3$ and (b) $BaHfS_3$.

Table S2: Calculated lattice parameters and respective deviations from experimental values.[4]

Compound	a (Å)	b (Å)	c (Å)
$BaZrS_3$	7.074 (+0.18%)	9.923 (−0.56%)	6.962 (−0.88%)
$BaHfS_3$	6.998 (−0.15%)	9.862 (−0.56%)	6.938 (−0.91%)

The density of states (DOS) and band structures were calculated using the HSE06 functional for both $BaZrS_3$ and $BaHfS_3$ and are presented in Figure S2. The DOS of $BaZrS_3$ indicates a band gap of 1.77 eV, aligning closely with experimental results of 1.75 eV[1] and 1.79 eV,[6] confirming the accuracy of the HSE06 functional.

The band structure in Figure S2b depicts the valence band in blue and the conduction band in orange. The regions of interest within the band structures are around the band edges, which dictate the band gap and are in agreement with the DOS plot. The valence band maximum (VBM) occurs at the Γ-point (0, 0, 0) in k-space. The occupied valence bands from 0 eV to −2 eV in $BaZrS_3$ lack dispersion as they are relatively flat, due to the localised S p states. The conduction band minimum (CBM) also lies on the Γ-point, identifying

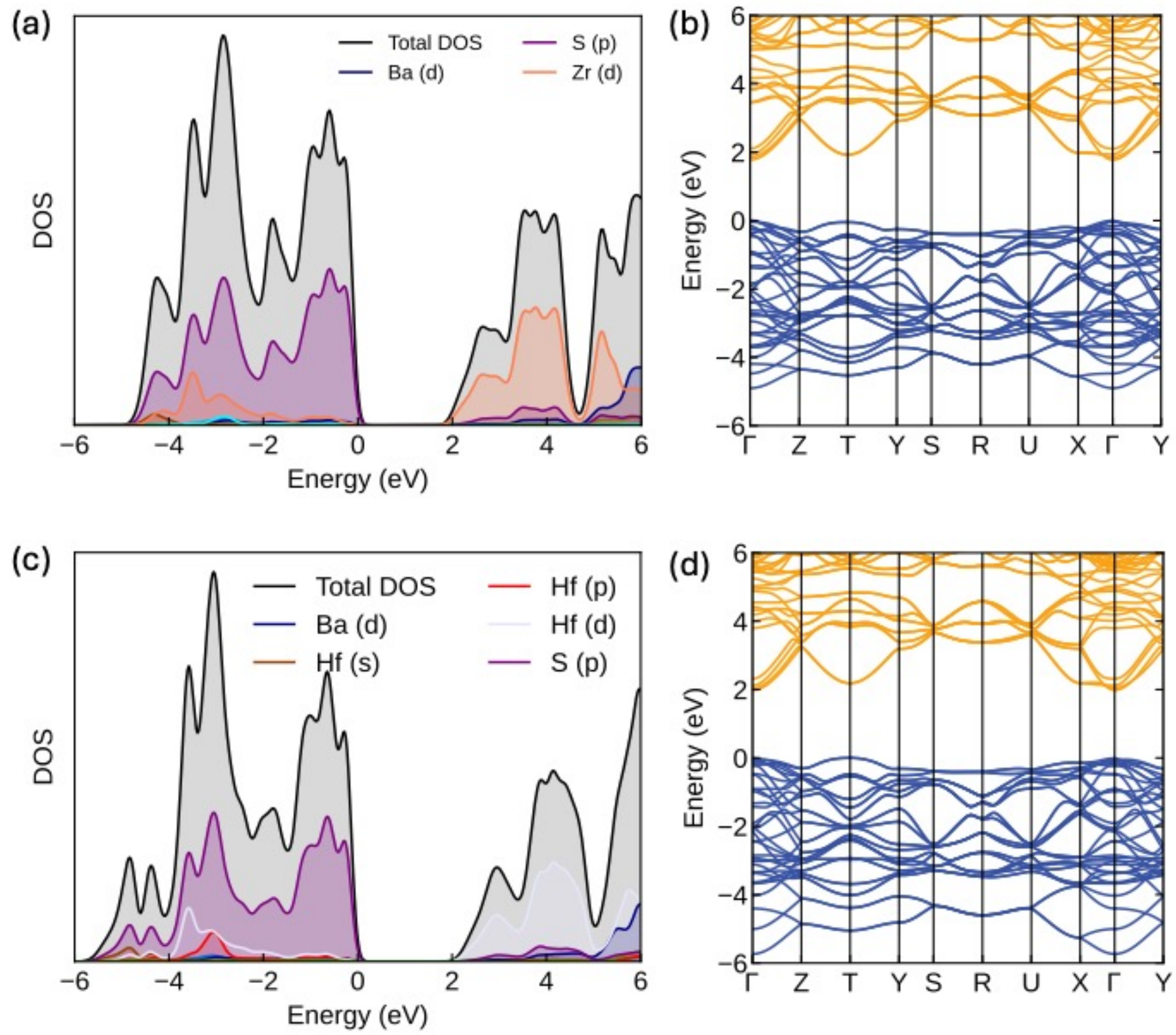

Figure S2: Partial density of states and band structure of (a–b) $BaZrS_3$ and (c–d) $BaHfS_3$.

$BaZrS_3$ as a direct band gap semiconductor. The CBM displays more dispersive bands at energy levels from 1.78–3 eV. This can be attributed to the broader DOS resulting from an overlap of Zr d-states with S p-states, leading to delocalisation and higher dispersion. The higher dispersion results in a lower electron effective mass (m_e) of $0.38\,m_0$ compared to a hole effective mass (m_h) of $0.48\,m_0$ in the less dispersed valence band, aligning well with the results of Huo et al.[7].

Figure S2c shows the DOS plot for $BaHfS_3$, where the band gap is slightly larger with a value of 1.98 eV, agreeing well with experimental band gaps of 2.06 eV.[5] The band structure in Figure S2d is similar to that of $BaZrS_3$, with strong dispersions in the CBM resulting in an electron effective mass of $0.29\,m_0$. The VB, on the other hand, exhibits a hole effective

mass of 2.41 m_0. This increase in m_h can be attributed to the VBM being located at the T-point rather than the Γ-point, making $BaHfS_3$ a slightly indirect semiconductor; however, it retains desirable absorption properties due to the presence of a direct transition at 1.99 eV at the Γ-point. Table S3 summarises the electronic properties of Ba(Zr,Hf)S_3.

Table S3: Optoelectronic properties of inorganic chalcogenide perovskites. The direct (E_g^d) and indirect (E_g^i) band gaps are given in eV, the effective masses (m_e and m_h) are given in units of the electron rest mass. We include the high-frequency dielectric constant (ε_∞) along the a, b, and c lattice vectors. The corresponding theoretical efficiency at 200 nm (η_{200}) is also provided.

	E_g^d	E_g^i	m_e	m_h	ε_∞			η_{200}
Compound	(eV)	(eV)	(m_0)	(m_0)	a	b	c	(%)
$BaZrS_3$	1.77	–	0.30	0.48	7.27	7.31	7.13	26.81
$BaHfS_3$	1.99	1.98	0.29	2.41	6.57	6.67	6.60	23.0

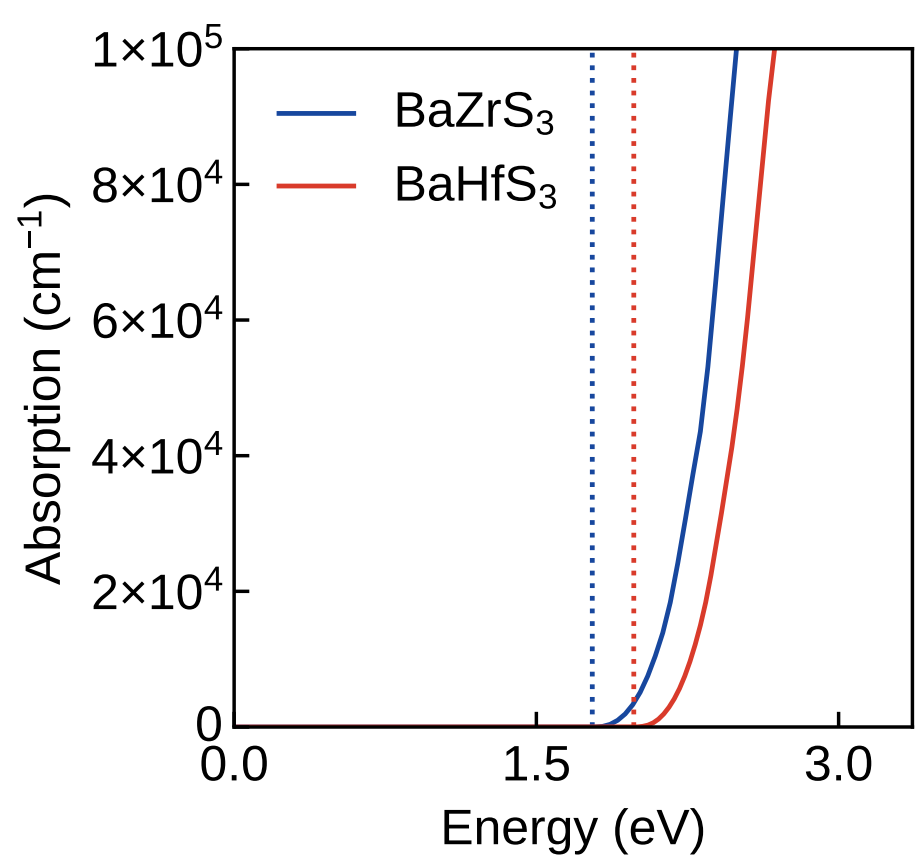

Figure S3: HSE06 calculated optical absorption spectra for $BaZrS_3$ and $BaHfS_3$ as a function of energy. Solid lines indicate the optical absorption and dashed lines indicate the fundamental band gap.

The absorption spectra for Ba(Zr,Hf)S_3 are shown in Figure S3. Both compounds exhibit strong absorption coefficients, represented by a sharp absorption edge which climbs to above 10^5 cm^{-1} within 0.5 eV of the onset, agreeing well with experimental literature.[2,4,8,9] Additionally, the high-frequency dielectric constants, ε_∞, and maximum theoretical efficiency

were calculated and are listed in Table S3 and Figure S4.

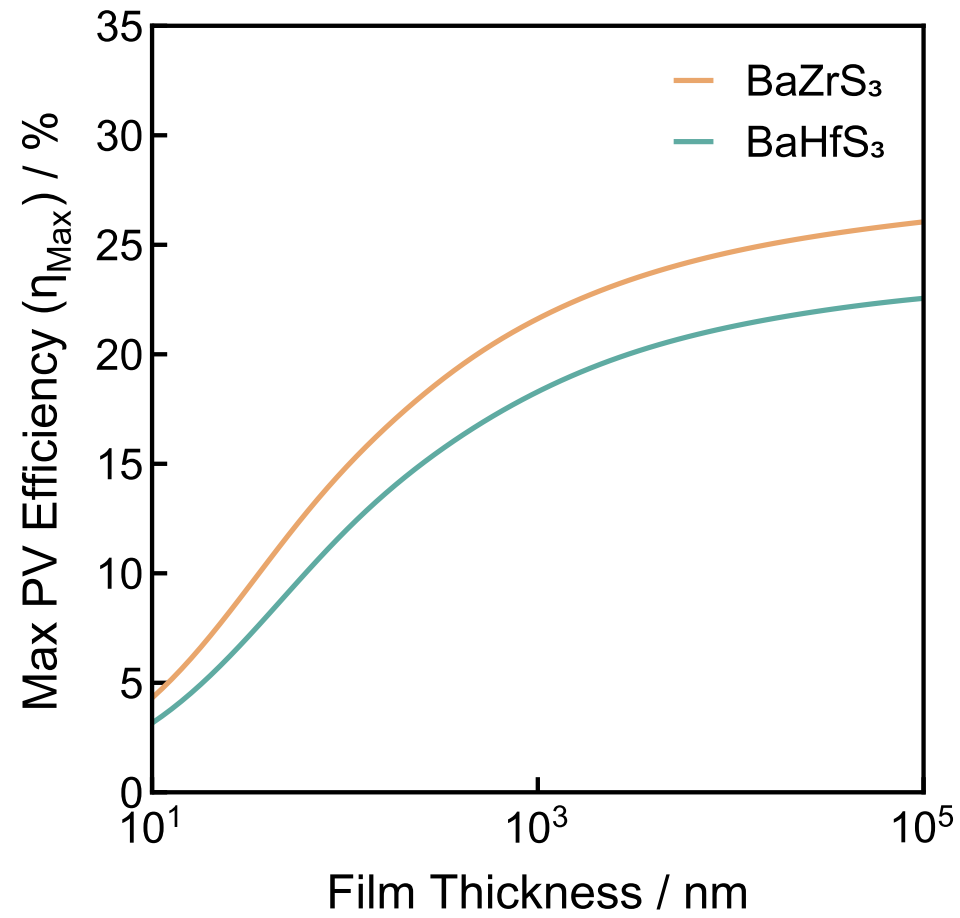

Figure S4: Maximum theoretical efficiency for $BaZrS_3$ and $BaHfS_3$ as a function of film thickness, calculated using HSE06.

S2 Materials Screening

Table S4: Tolerance factors (t) and octahedral factors (μ) for hybrid chalcogenide perovskites. Compounds that remain perovskite after relaxation are highlighted in bold.

Compound	R_A (pm)	R_B (pm)	R_X (pm)	t	μ
**$HZZrS_3$**	217	72	184	1.11	0.39
$AMZZrS_3$	267	72	184	1.25	0.39
**$HZZrSe_3$**	217	72	196	1.09	0.37
$AMZZrSe_3$	267	72	196	1.22	0.37
**$HZHfS_3$**	217	71	184	1.11	0.39
$AMZHfS_3$	267	71	184	1.25	0.39
**$HZHfSe_3$**	217	71	196	1.09	0.36
$AMZHfSe_3$	267	71	196	1.23	0.36
$AMSbS_3$	146	60	184	0.96	0.33
$HYSbS_3$	140	60	184	0.94	0.33
$MAMSbS_3$	216	60	184	1.16	0.33
$MASbS_3$	217	60	184	1.16	0.33
$HZSbS_3$	220	60	184	1.17	0.33
$HASbS_3$	216	60	184	1.16	0.33
$PHSbS_3$	212	60	184	1.15	0.33
$AZSbS_3$	238	60	184	1.22	0.33
$FASbS_3$	253	60	184	1.27	0.33
$EASbS_3$	274	60	184	1.33	0.33
$FOSbS_3$	258	60	184	1.28	0.33
$DMASbS_3$	272	60	184	1.32	0.33
$MHSbS_3$	275	60	184	1.33	0.33
$AZESbS_3$	250	60	184	1.26	0.33
$HMASbS_3$	275	60	184	1.33	0.33
$CASbS_3$	293	60	184	1.38	0.33
$TMASbS_3$	294	60	184	1.39	0.33
$PYSbS_3$	278	60	184	1.34	0.33
$TTMASbS_3$	292	60	184	1.38	0.33
$AMSbSe_3$	146	60	196	0.94	0.31
$HYSbSe_3$	140	60	196	0.93	0.31
$MAMSbSe_3$	216	60	196	1.14	0.31
$MASbSe_3$	217	60	196	1.14	0.31
$HZSbSe_3$	220	60	196	1.15	0.31
$HASbSe_3$	216	60	196	1.14	0.31
$PHSbSe_3$	212	60	196	1.13	0.31
$AZSbSe_3$	238	60	196	1.20	0.31

Continued on next page...

Table S4 – continued from previous page

Compound	R_A (pm)	R_B (pm)	R_X (pm)	t	μ
$FASbSe_3$	253	60	196	1.24	0.31
$EASbSe_3$	274	60	196	1.30	0.31
$FOSbSe_3$	258	60	196	1.25	0.31
$DMASbSe_3$	272	60	196	1.29	0.31
$MHSbSe_3$	275	60	196	1.30	0.31
$AZESbSe_3$	250	60	196	1.23	0.31
$HMASbSe_3$	275	60	196	1.30	0.31
$CASbSe_3$	293	60	196	1.35	0.31
$TMASbSe_3$	294	60	196	1.35	0.31
$PYSbSe_3$	278	60	196	1.31	0.31
$TTMASbSe_3$	292	60	196	1.35	0.31
$AMBiS_3$	146	76	184	0.90	0.41
**$HYBiS_3$**	140	76	184	0.88	0.41
$MAMBiS_3$	216	76	184	1.09	0.41
$MABiS_3$	217	76	184	1.09	0.41
$HZBiS_3$	220	76	184	1.10	0.41
$HABiS_3$	216	76	184	1.09	0.41
$PHBiS_3$	212	76	184	1.08	0.41
$AZBiS_3$	238	76	184	1.15	0.41
$FABiS_3$	253	76	184	1.19	0.41
$EABiS_3$	274	76	184	1.25	0.41
$FOBiS_3$	258	76	184	1.20	0.41
$DMABiS_3$	272	76	184	1.24	0.41
$MHBiS_3$	275	76	184	1.25	0.41
$AZEBiS_3$	250	76	184	1.18	0.41
$HMABiS_3$	275	76	184	1.25	0.41
$CABiS_3$	293	76	184	1.30	0.41
$TMABiS_3$	294	76	184	1.30	0.41
$PYBiS_3$	278	76	184	1.26	0.41
$TTMABiS_3$	292	76	184	1.29	0.41
$AMBiSe_3$	146	76	196	0.89	0.39
$HYBiSe_3$	140	76	196	0.87	0.39
$MAMBiSe_3$	216	76	196	1.07	0.39
$MABiSe_3$	217	76	196	1.07	0.39
$HZBiSe_3$	220	76	196	1.08	0.39
$HABiSe_3$	216	76	196	1.07	0.39
$PHBiSe_3$	212	76	196	1.06	0.39
$AZBiSe_3$	238	76	196	1.13	0.39
$FABiSe_3$	253	76	196	1.17	0.39
$EABiSe_3$	274	76	196	1.22	0.39
$FOBiSe_3$	258	76	196	1.18	0.39

Continued on next page...

Table S4 – continued from previous page

Compound	R_A (pm)	R_B (pm)	R_X (pm)	t	μ
$DMABiSe_3$	272	76	196	1.22	0.39
$MHBiSe_3$	275	76	196	1.22	0.39
$AZEBiSe_3$	250	76	196	1.16	0.39
$HMABiSe_3$	275	76	196	1.22	0.39
$CABiSe_3$	293	76	196	1.27	0.39
$TMABiSe_3$	294	76	196	1.27	0.39
$PYBiSe_3$	278	76	196	1.23	0.39
$TTMABiSe_3$	292	76	196	1.27	0.39

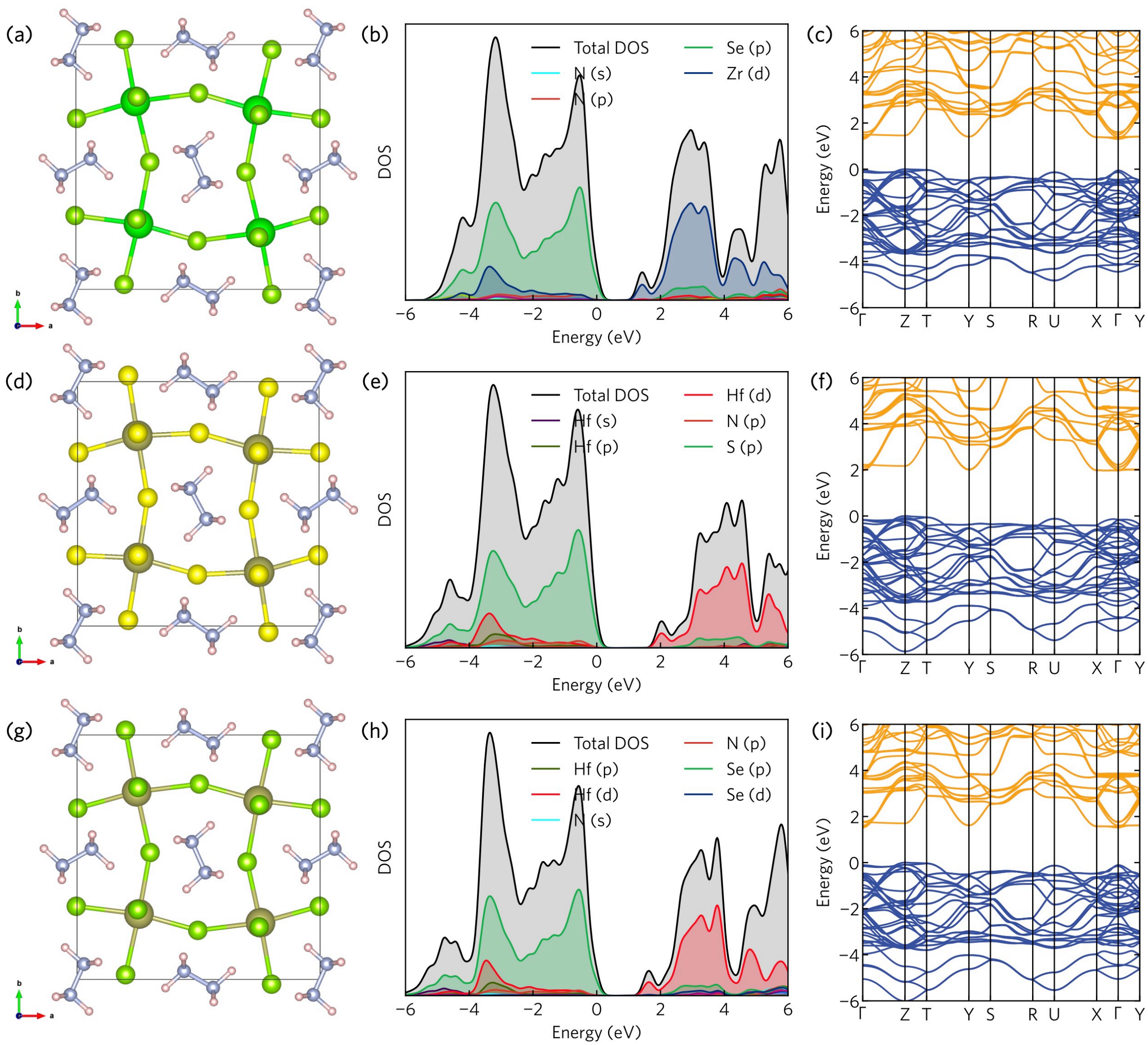

Figure S5: Atomic structures, partial density of states and band structures for (a–c) $HZZrSe_3$, (d–f) $HZHfS_3$ and (g–i) $HZHfSe_3$.

Table S5: The 46 compounds examined in this report with their converged k-points and phases formed after geometric optimisation and electronic convergence for the initial screening. Note, for the stable perovskites, the k-point mesh was reconverged following ground state structure determination from molecular dynamics annealing. The cut-off energy for all compounds was 500 eV.

ABS_3 perovskites			$ABSe_3$ perovskites		
Compound	k-point	Phase	Compound	k-point	Phase
$HYBiS_3$	$8 \times 8 \times 8$	Perovskite	$HYBiSe_3$	$8 \times 8 \times 8$	Non-perovskite
$AMBiS_3$	$9 \times 9 \times 9$	Non-perovskite	$AMBiSe_3$	$8 \times 8 \times 8$	Non-perovskite
$PHBiS_3$	$9 \times 9 \times 9$	Non-perovskite	$PHBiSe_3$	$9 \times 9 \times 9$	Non-perovskite
$HABiS_3$	$9 \times 9 \times 9$	Non-perovskite	$HABiSe_3$	$6 \times 6 \times 6$	Non-perovskite
$MABiS_3$	$6 \times 6 \times 6$	Non-perovskite	$MABiSe_3$	$6 \times 6 \times 6$	Non-perovskite
$HZBiS_3$	$7 \times 7 \times 7$	Non-perovskite	$HZBiSe_3$	$7 \times 7 \times 7$	Non-perovskite
$FABiS_3$	$9 \times 9 \times 9$	Non-perovskite	$FABiSe_3$	$10 \times 10 \times 10$	Non-perovskite
$FOBiS_3$	$8 \times 8 \times 8$	Non-perovskite	$FOBiSe_3$	$7 \times 7 \times 7$	Non-perovskite
$EABiS_3$	$7 \times 7 \times 7$	Non-perovskite	$EABiSe_3$	$7 \times 7 \times 7$	Non-perovskite
$AZBiS_3$	$7 \times 7 \times 7$	Non-perovskite	$AZBiSe_3$	$7 \times 7 \times 7$	Non-perovskite
$AZEBiS_3$	$8 \times 8 \times 8$	Non-perovskite	$AZEBiSe_3$	$9 \times 9 \times 9$	Non-perovskite
$DMABiS_3$	$7 \times 7 \times 7$	Non-perovskite	$DMABiSe_3$	$7 \times 7 \times 7$	Non-perovskite
$TMABiS_3$	$10 \times 10 \times 10$	Non-perovskite	$TMABiSe_3$	$5 \times 5 \times 5$	Non-perovskite
$TTMABiS_3$	$5 \times 5 \times 5$	Non-perovskite	$TTMABiSe_3$	$5 \times 5 \times 5$	Non-perovskite
$CABiS_3$	$8 \times 8 \times 8$	Non-perovskite	$CABiSe_3$	$8 \times 8 \times 8$	Non-perovskite
$PYBiS_3$	$6 \times 6 \times 6$	Non-perovskite	$PYBiSe_3$	$7 \times 7 \times 7$	Non-perovskite
$MHBiS_3$	$7 \times 7 \times 7$	Non-perovskite	$MHBiSe_3$	$7 \times 7 \times 7$	Non-perovskite
$MAMBiS_3$	$6 \times 6 \times 6$	Non-perovskite	$MAMBiSe_3$	$9 \times 9 \times 9$	Non-perovskite
$HMABiS_3$	$9 \times 9 \times 9$	Non-perovskite	$HMABiSe_3$	$9 \times 9 \times 9$	Non-perovskite
$HZZrS_3$	$6 \times 6 \times 6$	Perovskite	$HZZrSe_3$	$5 \times 5 \times 5$	Perovskite
$HZHfS_3$	$6 \times 6 \times 6$	Perovskite	$HZHfSe_3$	$5 \times 5 \times 5$	Perovskite
$AMZZrS_3$	$5 \times 5 \times 5$	Non-perovskite	$AMZZrSe_3$	$5 \times 5 \times 5$	Non-perovskite
$AMZHfS_3$	$6 \times 6 \times 6$	Non-perovskite	$AMZHfSe_3$	$5 \times 5 \times 5$	Non-perovskite

Table S6: Lattice parameters for the thermodynamically and dynamically stable chalcogenide perovskites calculated using the PBEsol functional.

Compound	a (Å)	b (Å)	c (Å)	α (°)	β (°)	γ (°)
$HZZrS_3$	9.9952	10.1295	5.0957	90	90	90
$HZZrSe_3$	10.4382	10.4845	5.3321	90	90	90
$HZHfS_3$	9.9445	10.0595	5.0581	90	90	90
$HZHfSe_3$	10.3763	10.4272	5.2987	90	90	90

References

(1) Gupta, T. et al. An Environmentally Stable and Lead-Free Chalcogenide Perovskite. *Advanced Functional Materials* **2020**, *30*, 2001387.

(2) Wei, X. et al. Realization of $BaZrS_3$ chalcogenide perovskite thin films for optoelectronics. *Nano Energy* **2020**, *68*, 104317.

(3) Mitzi, D.; Yan, Y. *High Performance Perovskite-Based Solar Cells (Final Technical Report)*; 2019.

(4) Nishigaki, Y.; Nagai, T.; Nishiwaki, M.; Aizawa, T.; Kozawa, M.; Hanzawa, K.; Kato, Y.; Sai, H.; Hiramatsu, H.; Hosono, H.; Fujiwara, H. Extraordinary Strong Band-Edge Absorption in Distorted Chalcogenide Perovskites. *Solar RRL* **2020**, *4*, 1900555.

(5) Hanzawa, K.; Iimura, S.; Hiramatsu, H.; Hosono, H. Material Design of Green-Light-Emitting Semiconductors: Perovskite-Type Sulfide $SrHfS_3$. *Journal of the American Chemical Society* **2019**, *141*, 5343–5349.

(6) Osei-Agyemang, E.; Koratkar, N.; Balasubramanian, G. Examining the Electron Transport in Chalcogenide Perovskite $BaZrS_3$. *Journal of Materials Chemistry C* **2021**, *9*, 3892–3900.

(7) Huo, Z.; Wei, S.-H.; Yin, W.-J. High-throughput screening of chalcogenide single perovskites by first-principles calculations for photovoltaics. *Journal of Physics D: Applied Physics* **2018**, *51*, 474003.

(8) Márquez, J. A.; Rusu, M.; Hempel, H.; Ahmet, I. Y.; Kölbach, M.; Simsek, I.; Choubrac, L.; Gurieva, G.; Gunder, R.; Schorr, S.; Unold, T. $BaZrS_3$ Chalcogenide Perovskite Thin Films by H_2S Sulfurization of Oxide Precursors. *The Journal of Physical Chemistry Letters* **2021**, *12*, 2148–2153.

(9) Qin, S. A promising chalcogenide perovskite-$BaZrS_3$-for application on tandem solar cells. *Applied and Computational Engineering* **2023**, *7*, 14–23.
